# Variational autoencoder enhanced analysis of energy metabolism and autophagy in exercising cardiomyocytes

**DOI:** 10.3389/ebm.2025.10489

**Published:** 2025-09-22

**Authors:** Liquan Chen, Yun Yang

**Affiliations:** ^1^ School of Culture and Tourism, Quzhou College of Technology, Quzhou, Zhejiang, China; ^2^ College of Physical Education, Henan Normal University, Xinxiang, China

**Keywords:** myocardial cell, energy autophagy, variational autoencoder, metabolite concentration, gene expression, feature extraction and analysis

## Abstract

Autophagy of myocardial cells involves the interaction of multiple molecular signaling pathways, and regulatory factors, while existing methods are difficult to handle. This study utilized the variational autoencoder (VAE) model to reveal the characteristic distribution of myocardial cell energy autophagy under different exercise conditions. First, this paper is based on mass spectrometry analysis, enzyme-linked immunosorbent assay ELISA (Enzyme-Linked Immunosorbent Assay) to determine the cardiomyocyte metabolite concentration data, and RNA-Seq (Ribonucleic Acid-Sequencing) to collect genes related to cardiomyocyte energy metabolism and autophagy expression data; in the VAE model, this paper utilizes the full connectivity layer to encode the data into potential representations, and reconstructs the numerical data through the numerical data decoder. The loss function is defined as the data reconstruction error and KL (Kullback-Leibler) scatter, and Adam is used to optimize the training process; the features are analyzed and the classification performance is verified under different motion conditions based on RF (Random Forest); the relationship between the features and metabolite concentration and gene expression is analyzed by LASSO (Least Absolute Shrinkage and Selection Operator) regression model to analyze the relationship between features and metabolite concentration and gene expression; the features in the latent space are downscaled using t-SNE (t-distributed Stochastic Neighbor Embedding) to visualize the feature distribution; finally, CRISPR-Cas9 (Clustered Regularly Interspaced Short Palindromic Repeats-Cas9) knockdown experiments to reveal the importance of AMPK, PGC1A, CPT1B, and SIRT1 in cardiomyocyte autophagy and energy metabolism, which provide potential targets for future gene-based therapies.

## Graphical Abstract

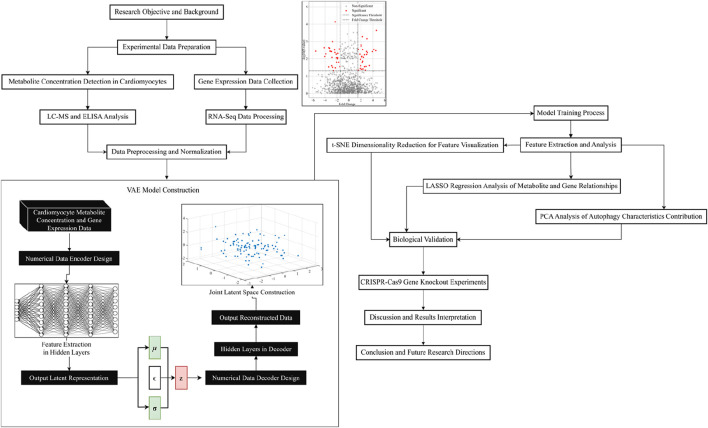

## Impact statement

This study advances our understanding of myocardial cell energy metabolism and autophagy, particularly under different exercise conditions. Using advanced technologies such as RNA sequencing, mass spectrometry, and machine learning models, the research identifies key molecular regulators involved in energy metabolism and autophagy, such as AMPK, PGC1A, CPT1B, and SIRT1. These findings provide new insights into how these factors interact to support cardiac function and reveal the importance of autophagy in maintaining heart cell health. By combining experimental and computational approaches, this work offers a deeper understanding of the regulatory networks that govern heart cell energy balance, which is crucial for developing targeted therapies for heart diseases. The results suggest that manipulating these key regulators could offer new therapeutic strategies for treating cardiovascular conditions, thereby making a significant contribution to the field of cardiovascular research and providing potential pathways for gene-based treatments.

## Introduction

Energy autophagy in cardiomyocytes is a complex and important biological process that plays a key role in maintaining cardiomyocyte function and responding to metabolic stress. Energy autophagy ensures normal cellular function by degrading and reusing damaged or excess intracellular components [1, 2]. The process involves the interaction of multiple molecular signaling pathways and regulatory factors [3, 4], and is particularly important in the pathogenesis of several cardiovascular diseases [5, 6]. Due to the high-dimensional complexity of the energy autophagy process, traditional research methods face many challenges in resolving its dynamic features and mechanisms [7, 8]. The development of new methods capable of handling high-dimensional data and revealing complex biological processes is of great research significance and practical value.

Nowadays, there are extensive studies in cardiomyocyte energy autophagy. In 2020, Fan Jiamao [9] et al. investigated the metabolite changes in cardiomyocytes under different metabolic stresses by enzyme-linked immunosorbent assay. However, this approach can only deal with data under a single dimension and cannot fully integrate and parse the complex interactions in multidimensional data [10, 11]. Meanwhile, traditional methods have certain limitations in data processing and feature extraction efficiency [12, 13]: although mass spectrometry analysis can provide detailed metabolite information, it is less efficient in dealing with complex datasets with large data volume and high dimensionality [14, 15]. In 2022, Wu Xun [16] et al. attempted to introduce SVM (Support Vector Machine) model to analyze metabolic data, and although some progress had been made, it was still deficient in the generalization and accuracy of high-dimensional data. Most of the past studies were limited to single-dimensional data analysis, which could not fully reveal the interactions between multidimensional data during cardiomyocyte energy autophagy, and were obviously insufficient for a comprehensive understanding of the complex mechanisms of the process.

To overcome the challenge of high dimensionality of data, in recent years, researchers have begun to explore the use of deep learning techniques to process and analyze complex biological data. Oriented towards the molecular mechanisms behind basic mitochondrial autophagy, Godtliebsen Gustav [17] et al. demonstrated that OXPHOS (oxidative phosphorylation) induction leaded to an increase in mitochondrial fragmentation through deep learning techniques. Evaluating cardiomyocytes from 289 childhood cancer survivors, Chaix Marie-A [18] et al. developed a risk prediction model incorporating genetic and clinical predictors using RF. Using deep learning for simulation analysis and *in vivo* validation, Iborra-Egea Oriol [19] et al. revealed the mechanism of action of Empagliflozin in Heart Failure with Reduced Ejection Fraction (HFrEF). In 2024, Liu Shuhui [20] et al. successfully extracted and analyzed the metabolic features of cancer cells using the VAE model, demonstrating the powerful ability of VAE in handling high-dimensional data. By encoding high-dimensional data into low-dimensional latent representations [21] and reconstructing the data through a decoder [22], the model effectively captures the main features and complex interactions in the data [23, 24]. The methods are able to discover hidden patterns and structures in the latent space while dealing with high-dimensional data [25, 26], effectively avoiding the complexity of directly dealing with high-dimensional data by means of low-dimensional representations of the latent space in order to capture the main features of the data [27, 28], and have obvious advantages in dealing with the nonlinear relationships and complex structures of the data [29, 30]. However, the application of VAE in cardiomyocyte energy autophagy characterization and analysis is still relatively rare until now, and further research and validation are urgently needed.

The application of VAE in extracting energy-autophagy features of myocardial cells offers unique advantages over PCA, UMAP, or standard autoencoders. One of the core features of VAE is its probabilistic encoding capability, which introduces a probability distribution in the latent space. This allows VAE not only to learn low-dimensional representations of the data but also to capture the uncertainty inherent in the data. Given that biological processes involve highly dynamic and nonlinear molecular interaction networks, the data often contain noise and heterogeneity. Through probabilistic encoding, VAE can generate smooth distributions in the latent space, thereby better reflecting the complex relationships between metabolite concentrations and gene expression.

Compared with traditional dimensionality reduction methods like PCA, VAE is capable of capturing nonlinear relationships, whereas PCA is limited to linear transformations. The energy metabolism and autophagy processes of myocardial cells involve multiple nonlinear regulatory pathways (such as interactions among AMPK, PGC1A, CPT1B, and SIRT1), making VAE more suitable for uncovering these intricate relationships. Unlike UMAP or t-SNE, which focus primarily on visualization, VAE can not only reduce dimensionality but also reconstruct the original data through its decoder, thereby validating the effectiveness of the latent features. While standard autoencoders can also perform nonlinear dimensionality reduction, they lack the probabilistic constraints of VAE, potentially leading to discontinuities in the latent space, which can affect the stability of subsequent analyses.

The aim of this paper is to extract and analyze the energy autophagy characteristics of cardiomyocytes under exercise conditions using the VAE model. Sixty healthy mice were selected and randomly divided into six groups, and the experimental groups were subjected to different types of exercise interventions, including resting condition, low-intensity exercise, moderate-intensity exercise, high-intensity exercise, prolonged low-intensity exercise, and intermittent high-intensity exercise. At the end of the exercise intervention, cardiomyocytes were isolated and extracted, metabolites were detected using LC-MS and ELISA, and gene expression analysis was performed. Cardiomyocyte metabolite concentrations and gene expression data were processed using the VAE model, and features were extracted and mapped to a low-dimensional potential space. The overall features of cardiomyocytes under different exercise intensities were found to be significantly different by t-SNE downscaling analysis. The distribution of features was more concentrated in the resting state and low-intensity exercise, while the features were more dispersed in the moderate- and high-intensity exercise groups, suggesting that high-intensity exercise triggered more metabolic pathways and intracellular state variability. The LASSO regression model further showed a strong relationship between exercise conditions and metabolite concentration and gene expression. The contribution of cardiomyocyte autophagy characteristics was analyzed using PCA (Principal Component Analysis), and gene knockout experiments were performed using CRISPR-Cas9 technology to observe the effect of specific genes on cardiomyocyte energy autophagy.

## Materials and methods

### Description of experimental data

To obtain cardiomyocyte data in this study, 60 healthy mice, half male and half female, weighing between 20 and 25 g were selected. The mice were randomly divided into 6 groups of 10 mice each and grouped according to exercise conditions as follows A-F:

A. Resting state group: no exercise intervention.
B. Low-intensity exercise group: mice ran on a low-speed rotor for 30 min.
C. Medium intensity exercise group: mice ran on a medium speed rotor for 45 min.
D. High-intensity exercise group: mice are subjected to high-intensity interval training, which consist of alternating short bursts of high-velocity running and resting for 20 min.
E. Prolonged low-intensity exercise group: mice ran continuously for 90 min on a low-speed rotor.
F. Intermittent high-intensity exercise group: mice are subjected to high-intensity interval training, with each cycle consisting of 4 min of high-intensity running and 2 min of low-intensity slow walking, lasting a total of 24 min.

### Measurement of cardiomyocyte metabolite concentration data

Immediately after the end of the exercise intervention, the mice were euthanized to ensure tissue sample activity. Mouse hearts were rapidly collected for cardiomyocyte isolation and extraction. Cardiomyocytes were washed with saline to remove blood and other impurities, cell samples were rapidly frozen by liquid nitrogen and stored in a refrigerator at −80°C to ensure that the samples did not degrade. The frozen cardiomyocyte samples were thawed to 4°C, and metabolite extraction was performed by adding a solvent mixture of methanol/water/chloroform (2:2:1), vortexing, centrifuging the supernatant, and collecting the metabolite extracts to avoid interference from cellular debris.

The extracted metabolites were concentrated and dried and redissolved in 50% aqueous methanol solution. The different types of metabolites were detected separately by positive and negative ion mode using Liquid Chromatography-Mass Spectrometry (LC-MS). The mass spectrometry data were acquired into a computer, and peak detection, peak alignment and normalization were performed using the mass spectrometry data processing software MetaboAnalyst to obtain the relative concentration data of the metabolites.

At the same time, pre-treatment and sample preparation based on ELISA were performed: a series of standard samples with different concentrations were prepared for the establishment of concentration standard curves of metabolites, and cardiomyocyte samples and standard samples were loaded into the wells of ELISA plates pre-coated with antigens, respectively. Specific enzyme-labeled antibodies were added to bind to the antigen and washes were performed to remove non-specific binding material. Substrate was added to produce a color reaction and absorbance was measured using an enzyme marker to determine metabolite concentration.

### Cardiomyocyte energy metabolism, autophagy-related gene expression data Collection

The same cardiomyocyte samples were collected as those for metabolite analysis, and total RNA was extracted using TRIzol reagent to ensure the integrity and purity of the RNA samples, and the concentration and quality of the RNA were assessed using NanoDrop and Bioanalyzer (RNA Integrity Number >7).

Ribosomal RNA from total RNA was removed using the rRNA (Ribosomal RNA) Removal Kit to obtain mRNA (Messenger RNA). Using the mRNA-Seq (Messenger RNA Sequencing) Library Construction Kit to reverse transcribe the mRNA into cDNA (Complementary DNA), which was fragmented, spliced, and amplified by PCR (Polymerase Chain Reaction) to construct an RNA-Seq sequencing library.

The constructed RNA-Seq library was sent to NovaSeq, a high-throughput sequencing platform, for sequencing. The raw data obtained from sequencing was collected and quality controlled using FastQC (Fast Quality Control) to remove low-quality reads and splice contamination.

High-quality sequencing reads were compared to the reference genome based on HISAT2 (Hierarchical Indexing for Spliced Alignment of Transcripts 2), and the results were quantified using FeatureCounts comparisons to get the read counts for each gene, which DESeq2 normalized to normalization to obtain the expression TPM of each gene under different exercise conditions.

**FIGURE 1:**
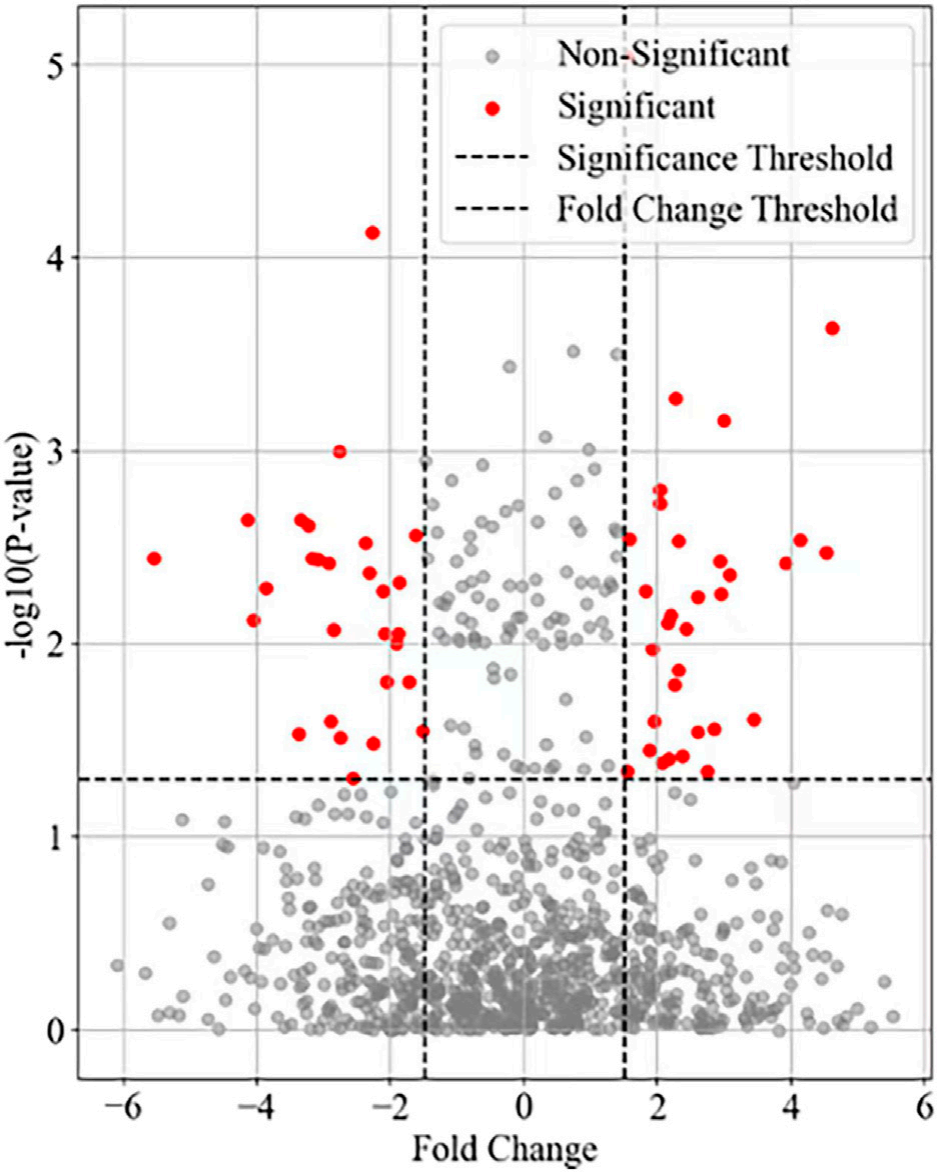
Differential expression of autophagy related genes in myocardial cells.

The research design divided 60 mice into 6 groups (a resting state group and 5 exercise intervention groups), detected the gene expression data of myocardial cells in each group through RNA-Seq technology, and used a volcano plot to visualize the differentially expressed genes between each experimental group (Groups B-F) and the resting group (Group A). The results of visualizing the gene variance analysis based on the volcano map are shown in Figure 1.

**FIGURE 2:**
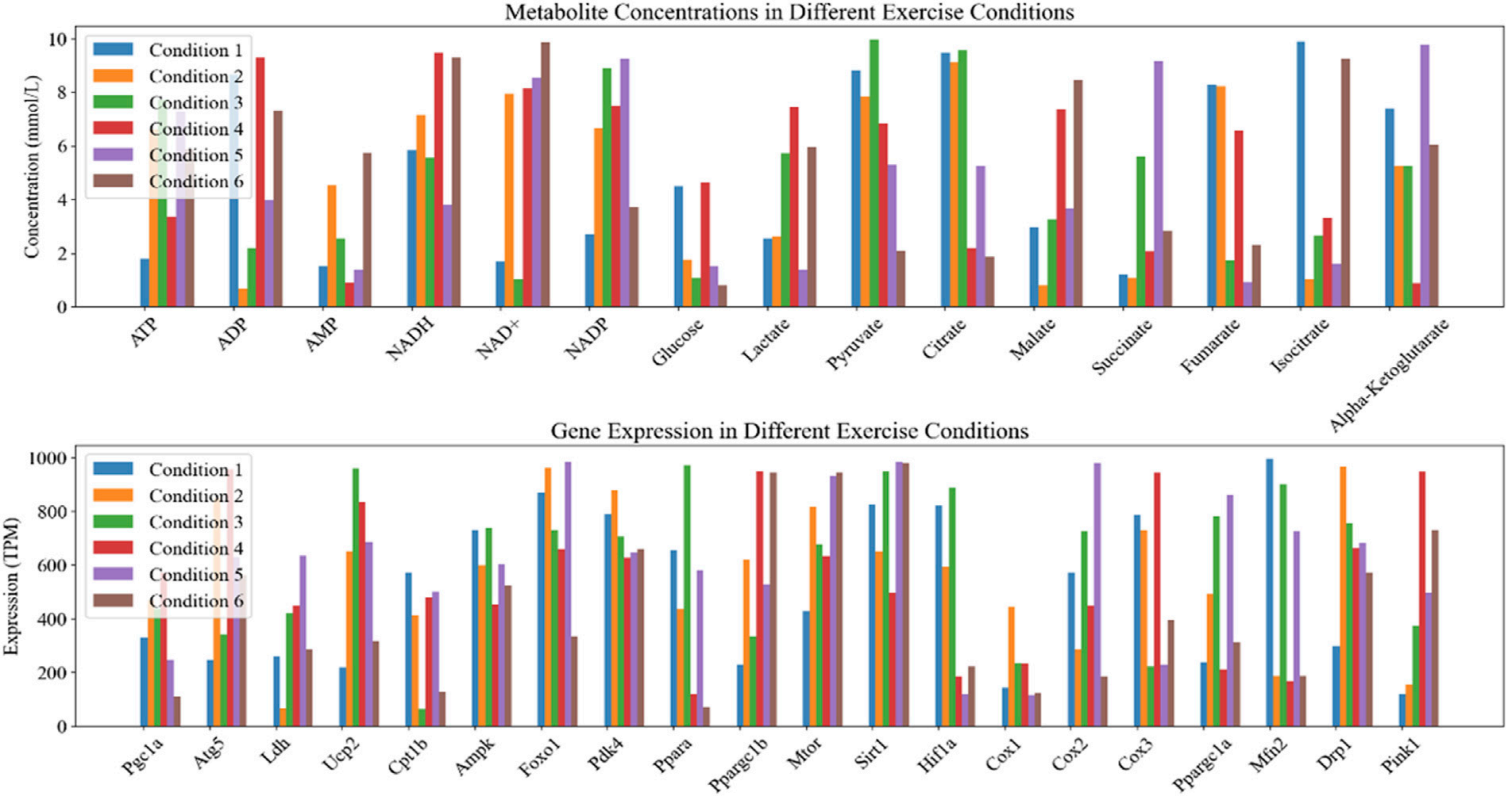
Statistics of changes in metabolic product concentration and gene expression in myocardial cells.

The horizontal axis in Figure 1 represents Fold Change, while the vertical axis represents the negative logarithmic value of statistical significance (- log10 (P-value)). Each data point represents a gene, and the fold-change and significance P-value of the data points are measures of difference. Top20 genes were selected as the focus of the study. The mean values of metabolite concentrations and gene expression changes in mouse cardiomyocytes under the corresponding experimental groups are shown in Figure 2.

In Figure 2, Condition 1-6 corresponds to experimental groups A-F. The upper graph demonstrates the changes of metabolite concentrations under different exercise conditions, the horizontal axis indicates different metabolites, and the vertical axis indicates product concentrations (unit: mmol/L). The lower graph shows the changes of gene expression under different exercise conditions, the horizontal axis indicates different genes, and the vertical axis indicates gene expression (unit: TPM/Transcripts Per Million).

Among them ATP, ADP, AMP are the core molecules of intracellular energy metabolism [31, 32]. NADH, NAD+, NADP are important coenzymes in redox reactions and energy metabolism [33]. Glucose, Lactate, Pyruvate are the key products of glucose metabolism, which are associated with the provision of energy supply by cardiomyocytes [34]. Citrate, Malate, Succinate, Fumarate, Isocitrate, Alpha-Ketoglutarate are key intermediates in the citric acid cycle and are involved in energy production and metabolic pathways [35]. Among the gene expression types, genes such as Pgc1a, Atg5, Ldh, Ucp2, Cpt1b, Ampk, Foxo1, Pdk4, Ppara, Ppargc1b, Mtor, Sirt1, and Hif1a are associated with the processes of energy metabolism, autophagy, and oxidative stress. Cox1, Cox2, Cox3, Mfn2, Drp1, Pink1 and other genes are involved in mitochondrial function, apoptosis and mitochondrial dynamics processes [36, 37].

Genes such as AMPK, PGC1A, CPT1B, and SIRT1 play central regulatory roles in cellular metabolism and autophagy mechanisms. They interact through complex signaling networks to collectively maintain energy homeostasis and autophagic balance in cardiomyocytes. AMPK acts as a cellular energy sensor, becoming activated when ATP levels decrease. It promotes catabolic pathways (such as fatty acid oxidation and glycolysis) by phosphorylating downstream targets to restore energy supply while inhibiting anabolic processes. Additionally, AMPK can directly or indirectly regulate the expression of autophagy-related genes (e.g., Atg5), thereby enhancing autophagic activity to respond to energy crises. PGC1A is a key regulator of mitochondrial biogenesis and function, working synergistically with various transcription factors (e.g., Ppara and Foxo1) to modulate the expression of genes associated with fatty acid oxidation, the electron transport chain, and oxidative phosphorylation, such as Cox1, Cox2, and Cpt1b, optimizing the energy production efficiency of cardiomyocytes. CPT1B is the rate-limiting enzyme for long-chain fatty acids entering mitochondria for β-oxidation, whose expression is regulated by PGC1A and Ppara, directly influencing the ability of cardiomyocytes to utilize fatty acids. SIRT1 is an NAD+-dependent deacetylase that senses changes in the intracellular NAD+/NADH ratio to regulate downstream target genes closely related to energy metabolism and autophagy (e.g., Pgc1a and Foxo1). Under stress conditions, it enhances autophagy pathway activity through deacetylation modifications. These genes not only play critical roles in their respective functional modules but also form tightly interconnected networks through cross-regulation. For example, AMPK can activate SIRT1 activity, while SIRT1 can reciprocally regulate the AMPK signaling pathway. This bidirectional feedback mechanism ensures the adaptive response capability of cardiomyocytes under different exercise conditions.

## VAE model construction

**FIGURE 3:**
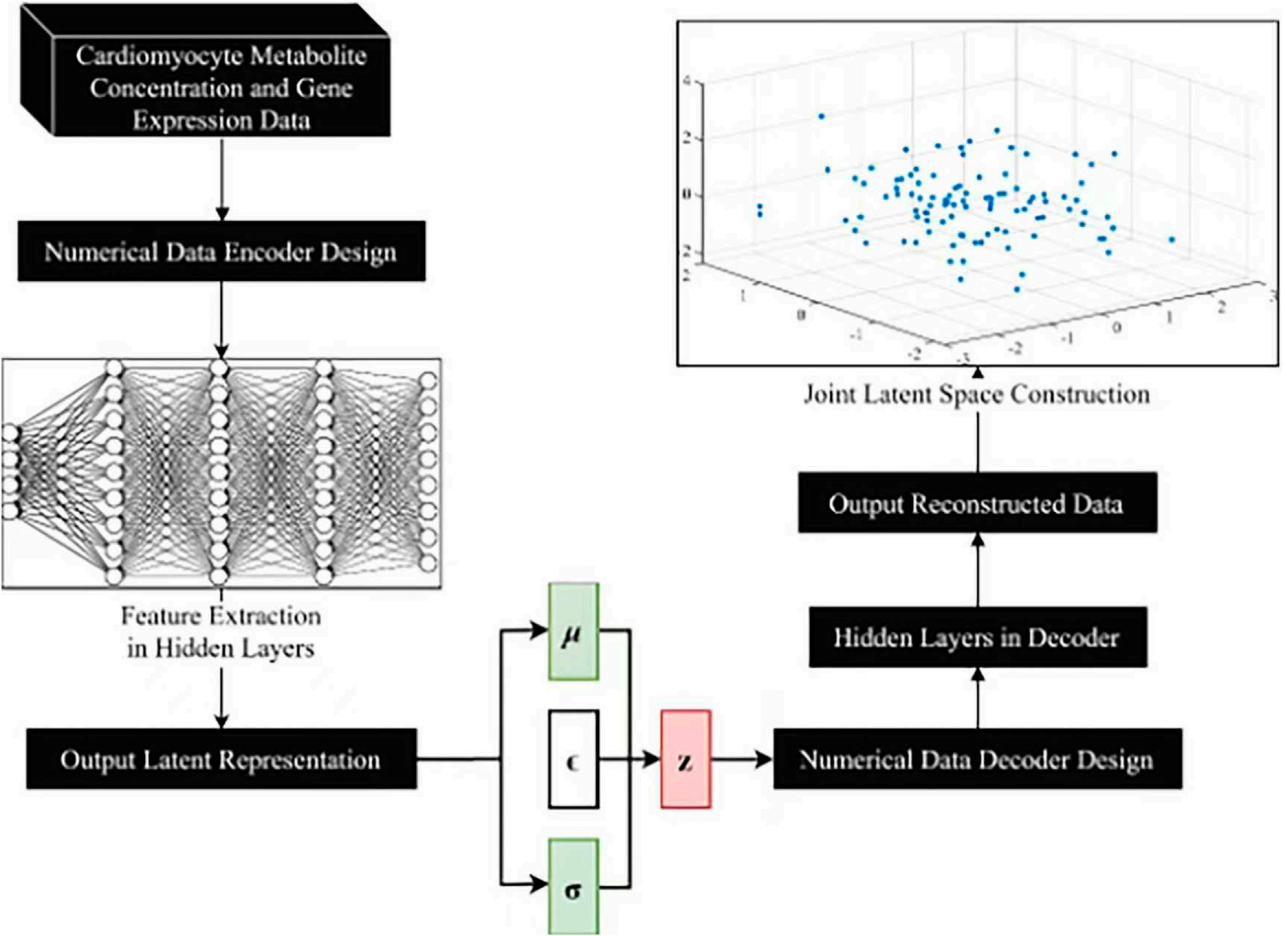
VAE model design.

The overall design for the VAE model is shown in Figure 3. The numerical data encoder receives cardiomyocyte metabolite concentration data and gene expression data as input. The features are gradually extracted through multiple fully connected layers to capture the complex correlations of the data and map the input data into a low-dimensional joint potential space. A symmetric decoder structure is designed to match the encoder to accurately reconstruct the original data, and the decoder parameters are optimized by back propagation algorithm to minimize the reconstruction error.

Before inputting high-dimensional omics data into the VAE model, preprocessing of transcriptomic and metabolomic data was performed to ensure data quality and compatibility for subsequent analyses. In this study, a logarithmic transformation was applied to the metabolite concentration data to mitigate the impact of its skewed distribution, which commonly occurs in metabolomic datasets due to the wide dynamic range of metabolite concentrations. This transformation not only stabilizes the data variance but also reduces the dominance of highly abundant metabolites, resulting in a more balanced representation of the data. For the gene expression data, z-score normalization was used to standardize the expression values across different genes.

To correct for batch effects, the widely used empirical Bayes framework, ComBat was employed. This method adjusts for batch-specific biases while preserving biological variability, thereby enhancing the reliability and reproducibility of downstream analyses. Additionally, statistical methods such as principal component analysis (PCA) and hierarchical clustering were utilized for outlier detection to identify and exclude samples that deviate significantly from the overall data distribution.

### Numerical data encoder design

The numerical data encoder adopts a deep neural network structure, and its inputs are cardiomyocyte metabolite concentration data x and expression-based data y , i.e., $x\in R^{n\times m_{x}},y\in R^{n\times m_{y}}$ , where n denotes the number of samples, and $m_{x}$ and $m_{y}$ denote the metabolite and gene dimensions, respectively.

The encoder objective is to map the input data to a Gaussian distribution parameter $\left(\mu ,\log\sigma^{2}\right)$ in the latent space, where μ and σ are the mean and standard deviation vectors of the latent space, and the encoder structure denoted as show in Equation 1:

$$\mu ,\log\sigma^{2}=f_{\text{encoder}}\left(x,y;\theta_{\text{encoder}}\right)$$ (1)

Here, $f_{\text{encoder}}$ denotes the mapping function of the encoder and $\theta_{\text{encoder}}$ denotes the encoder parameters. The encoder uses a multilayer neural network structure, where the output $h_{i}$ of layer i denoted as show in Equation 2:

$$h_{i}=\sigma \left(W_{i}\left[h_{i-1},x,y\right]+b_{i}\right)$$ (2)

Among them, $W_{i}$ and $b_{i}$ denote the weights and bias parameters of the ith layer respectively, σ denotes the activation function, and $\left[h_{i-1},x,y\right]$ denotes connecting the output of the previous layer with the input data. The final encoder achieves efficient coding of the input data by mapping the input data to the Gaussian distribution parameters in the latent space.

### Numerical data decoder design

The numerical data decoder aims to decode the latent representation z into raw data. The goal of the decoder is to generate a conditional probability distribution $p\left(t|u\right)$ of the raw data given the latent variable u , where t represents the raw data. To approximate this conditional distribution, the decoder maps latent variables u to reconstructed data $t^{\prime }=f_{\text{decoder}}\left(u;\theta_{\text{decoder}}\right)$ in the data space, where $f_{\text{decoder}}$ denotes the decoder mapping function and $\theta_{\text{decoder}}$ denotes the decoder parameters.

The decoder uses a deep neural network structure and the computation of its output $t^{\prime }$ is represented by Equations 3, 4:

$$H_{i}=\sigma \left(W_{i}h_{i-1}+b_{i}\right)$$ (3)

$$t^{\prime }=\text{sigmoid}\left(W^{\prime }h_{L}+b^{\prime }\right)$$ (4)

Here, $H_{i}$ denotes the hidden state of the decoder, $W^{\prime }$ and $b^{\prime }$ denote the weight and bias parameters of the last layer, $h_{L}$ denotes the hidden state of the last layer of the decoder and sigmoid denotes the activation function.

By optimizing the parameters, the decoder accurately reconstructs the input data.

### Joint potential space construction

A shared joint latent space is formed by combining the latent representations of the encoder and decoder for information transfer and exchange. The joint latent variable z is introduced and its conditional distribution is represented in Equation 5:

$$q\left(z|x,y\right)=N\left(\left.z\right|\mu_{\text{encoder}}\left(x,y\right),\Sigma_{\text{encoder}}\left(x,y\right)\right)$$ (5)

Among them, $\mu_{\text{encoder}}\left(x,y\right)$ and $\Sigma_{\text{encoder}}\left(x,y\right)$ denote the mean vector and covariance matrix of the latent variables output by the encoder, respectively. To realize the information transfer between the encoder and decoder, the variational inference technique is introduced to decompose the joint latent variable into the output of the encoder and the input of the decoder, i.e., $z=\mu_{\text{encoder}}\left(x,y\right)+\epsilon ⊙\sigma_{\text{encoder}}\left(x,y\right)$ , where ϵ denotes a random variable that obeys a standard normal distribution, and ⊙ denotes an element-by-element multiplication.

The training process is defined by maximizing the variational lower bound, as shown in Equation 6, to optimize the model parameters.

$$L=E_{q\left(z|x,y\right)}\left[\log p\left(x^{\prime }\left|z\right.\right)\right]-D_{\text{KL}}\left[q\left(z|x,y\right)\left\|p\right.\left(z\right)\right]$$ (6)

Where, the first term is the reconstruction loss, which measures the difference between the reconstructed data $x^{\prime }$ and the original data x , and the second term is the KL scatter, which measures the difference between the distribution of latent variables in the output of the encoder $q\left(z|x,y\right)$ and the *a priori* distribution $p\left(z\right)$ .

Effective information transfer and joint potential space construction between the encoder and decoder is achieved by optimizing the variational lower bound for effective representation and reconstruction of the input data.

## Model training process

During the model training process, the cardiomyocyte metabolite concentration data and the gene expression data were paired to form a joint dataset so that the encoder and decoder could process both types of data simultaneously. Defining the loss function to supervise the model training process, which consists of 2 components: data reconstruction error and KL scatter.

The Root Mean Square Error (RMSE) measure of reconstruction error is calculated using Equation 7.

$$Reconstruction\;Loss=\sqrt{\frac{1}{n}\sum_{i=1}^{n}{\left(x_{i}-x_{i}^{\prime }\right)}^{2}}$$ (7)

Here, n denotes the number of samples, and $x_{i}$ and $x_{i}^{\prime }$ denote the original and reconstructed data of the ith sample, respectively. The KL dispersion is calculated according to Equation 8.

$$D_{\text{KL}}\left[q\left(z|x,y\right)\left\|p\right.\left(z\right)\right]=\frac{1}{2}\sum_{i=1}^{k}\left(\sigma_{i}^{2}+\mu_{i}^{2}-\log\sigma_{i}^{2}-1\right)$$ (8)

**FIGURE 4:**
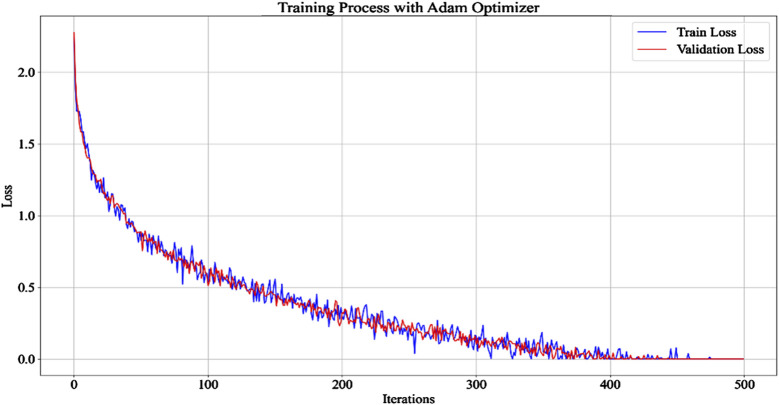
Model iteration process.

Among them, k denotes the dimension of the latent variable, and $\mu_{i}$ and $\sigma_{i}$ denote the mean and standard deviation of the latent variable output by the encoder, respectively. During the training process, the model parameters are updated to minimize the loss function using the Adam optimizer, which is an adaptive learning rate optimization algorithm that adaptively adjusts the learning rate according to the parameter gradient to accelerate the model convergence process. The gradient of the loss function with respect to the model parameters is calculated by the back-propagation algorithm, and the Adam optimizer is used to update the model parameters, which makes the loss function decrease gradually, so that the VAE model learns effective data representation and reconstruction laws. The process is shown in Figure 4.

In Figure 4, the VAE model is shown in 500 iterations, with the number of iterations on the x-axis and the loss values on the y-axis. The model converges after 400 iterations and remains constant after 477 iterations. The similarity in the trend of loss values in the training and validation sets indicates that the model has good generalization ability during training. The operational efficiency and complexity of the model’s encoding and decoding were further validated, with results shown in Table 1.

**TABLE 1:** Model encoding and decoding efficiency.

Index	Numerical data encoder	Numerical data decoder	Joint latent space construction
Computation Time (seconds/epoch)	0.85	1.2	0.45
Number of Parameters (millions)	12.5	15.3	8.7
Memory Usage (GB)	6.2	7.8	4.5
Data Dimensionality (Input → Output)	(n × m_x + n × m_y) → (n × k)	(n × k) → (n × m_x + n × m_y)	(n × k)

Table 1 shows the operational efficiency and complexity of the model’s encoding and decoding. It can be seen that the computation time for the numerical data encoder is only 0.85 s per epoch, with 12.5 million parameters and a memory usage of 6.2 GB. The input dimension is (n × m_x + n × m_y), which is mapped to a low-dimensional latent space (n × k), demonstrating its high efficiency in handling dimensionality reduction of high-dimensional data. On the other hand, the numerical data decoder, which needs to reconstruct the low-dimensional latent representation (n × k) back into high-dimensional original data (n × m_x + n × m_y), has an increased computation time and memory usage to 1.2 s per epoch and 7.8 GB respectively, with the number of parameters reaching 15.3 million, indicating a higher demand for computational resources during the decoding process. The construction of the joint latent space, however, exhibits higher efficiency, with a computation time of just 0.45 s per epoch, 8.7 million parameters, and a memory usage of 4.5 GB. It also maintains a low-dimensional output (n × k). By integrating and transferring information through a low-dimensional latent space, it effectively reduces complexity and ensures a balanced overall performance of the model.

## Feature extraction and analysis

Random Forest classifier is used to analyze the features in different sports conditions by constructing multiple decision trees and synthesizing the results for classification. The accuracy, precision, recall, and F1 score are calculated to quantify the effectiveness of the features for distinguishing different sports conditions, and the results are shown in Table 2.

**TABLE 2:** Classification results of energy autophagy characteristics of exercise myocardial cells.

Experimental group	Accuracy	Precision	Recall	F1 score
A	0.85	0.90	0.90	0.90
B	0.94	0.94	0.92	0.93
C	0.89	0.90	0.94	0.92
D	0.96	0.87	0.88	0.87
E	0.90	0.85	0.90	0.87
F	0.88	0.83	0.83	0.83

**FIGURE 5:**
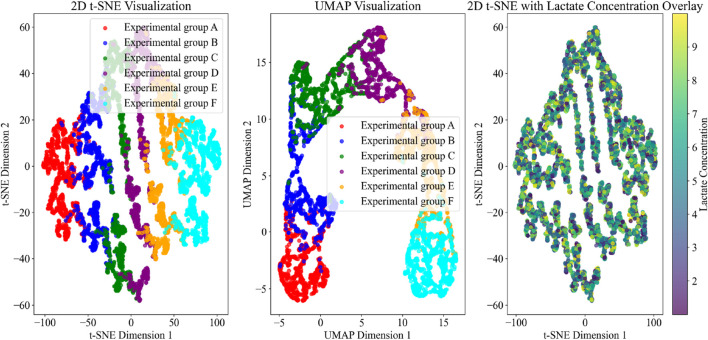
Dimensionality reduction and lactate overlay of cardiomyocyte features under exercise conditions.

As can be seen from Table 2, the model reaches high classification performance for different experimental groups (A-F), with all indicators above 0.83, indicating that the energy autophagy features of exercising cardiomyocytes have a high degree of differentiation under different exercise intensities. The features in the potential space of the VAE model are downscaled by the t-SNE algorithm. Setting the perplexity degree to 30, which makes the local structure more prominent, i.e., the clustering between similar samples is better; the number of iterations is 1000, to ensure that the algorithm has enough time to converge to a stable layout. The two-dimensional feature distribution map in potential space was obtained to observe the feature distribution of cardiomyocyte energy autophagy and its change trend under different exercise conditions, as shown in Figure 5.

Figure 5 uses t-SNE, UMAP, and lactate concentration overlay visualization methods to display the energy metabolism and autophagy characteristics of cardiomyocytes under different exercise conditions. The t-SNE results clearly distinguish the distribution patterns of each experimental group, particularly highlighting the significant separation of experimental group F under high-intensity exercise, reflecting its unique metabolic and autophagic state. UMAP further optimizes the presentation of global structure, emphasizing the boundaries between experimental groups, especially showing smoother cluster distributions in moderate-intensity exercise groups (such as B, C, and D). The t-SNE plot with lactate concentration overlay reveals a gradient change in lactate levels as exercise intensity increases, with high-lactate regions concentrated in experimental group F, indicating significantly enhanced anaerobic metabolism during high-intensity exercise.

In this study, the determination of the L1 regularization parameter value of 0.01 was based on the need to balance model performance and sparsity. Multiple experiments were conducted on the training dataset, and cross-validation was used to evaluate the impact of different regularization parameter values (e.g., 0.001, 0.01, 0.1, 1) on model performance. When the regularization parameter value was too small (e.g., 0.001), the model failed to effectively perform feature selection, resulting in the retention of too many irrelevant or redundant features, which increased model complexity and the risk of overfitting. Conversely, when the regularization parameter value was too large (e.g., 0.1 or 1), too many feature coefficients were compressed to zero, oversimplifying critical information and leading to underfitting. After systematic comparison, it was found that when the regularization parameter value was set to 0.01, the model could maintain a high prediction accuracy while selecting features closely related to exercise conditions, metabolite concentrations, and gene expression.

**FIGURE 6:**
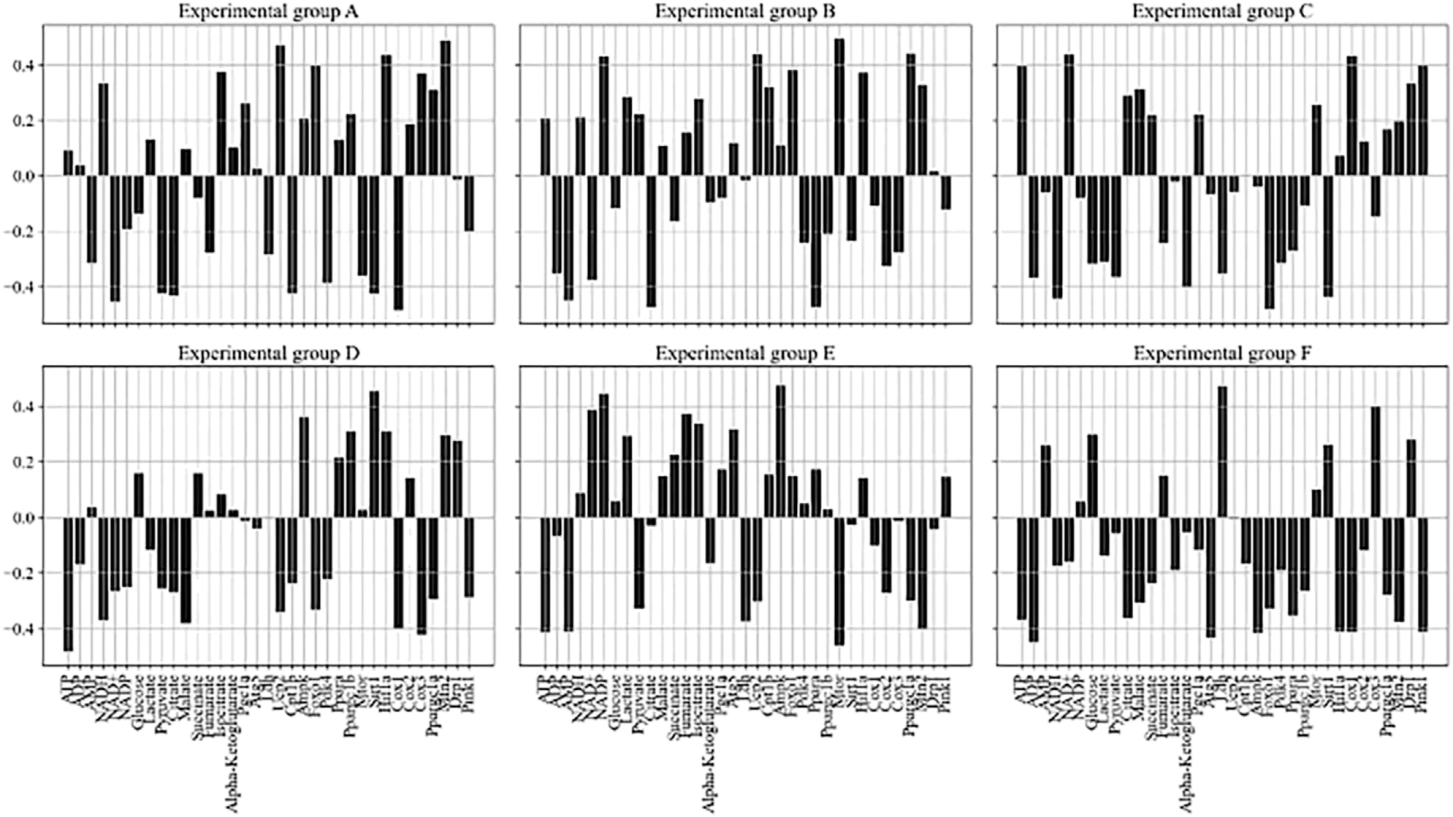
Regression coefficients between each exercise condition and metabolite concentration, gene expression. **(A)** Resting state group. **(B)** Low-intensity exercise group. **(C)** Moderate-intensity exercise group. **(D)** High-intensity exercise group. **(E)** Prolonged low-intensity exercise group. **(F)** Intermittent high-intensity exercise group.

The LASSO regression model was further used to explore the relationship between exercise conditions and changes in metabolite concentrations and gene expression characteristics. Feature selection and model sparsification were achieved by penalizing larger coefficients through the introduction of an L1 regularization term. The regularization parameter was set to 0.01 in the study and the model was fitted using the training dataset. After the fitting was completed, the regression coefficients between each exercise condition and metabolite concentration and gene expression were obtained to reveal the extent and direction of the effect of exercise conditions on the target variables in the experimental group, and the results are shown in Figure 6.

In Figure 6, six subgraphs represent different experimental groups (A-F), with the concentration of each metabolite and gene expression characteristics in the horizontal direction, and the regression coefficients of the model in the vertical direction. ATP, as an important carrier of energy, is closely related to the activation/inhibition of metabolic pathways with the change of its concentration under different exercise conditions. Exercise condition B significantly increased the concentration of ATP (0.26), indicating that this condition increased energy demand and promoted the synthesis of energetic substances. In contrast, exercise condition D showed a significant decrease in ATP concentration (−0.48), reflecting increased energy expenditure and accelerated ATP catabolism due to high-intensity prolonged exercise. Changes in gene expression also provided insight into how exercise affected intracellular homeostasis. Pgc1a showed higher expression levels (0.26) under exercise condition A, which correlated with enhanced mitochondrial function and increased efficiency of energy production, which in turn adapted to the changes in energy demand brought about by exercise.

At the same time, the interactions between metabolites and gene expression do not exist in isolation but constitute a complex network. The concentration of Lactate decreases (−0.31) accompanied by an increase in the expression of Pgc1a (0.22) under exercise condition C, suggesting that the reduction of lactate stimulates mitochondrial biogenesis and increases the efficiency of oxidative phosphorylation in response to the energy demands of the hypoxic conditions. The relatively high expression level of Hif1a (0.14) under exercise condition E, when ATP concentration is low (−0.41), reflects the fact that cells in a hypoxic environment promote adaptive responses including an increase in glycolytic activity by up-regulating Hif1a to meet energy demands. Exercise can remodel metabolic pathways over time by regulating gene expression to adapt to different physiological demands.

**FIGURE 7:**
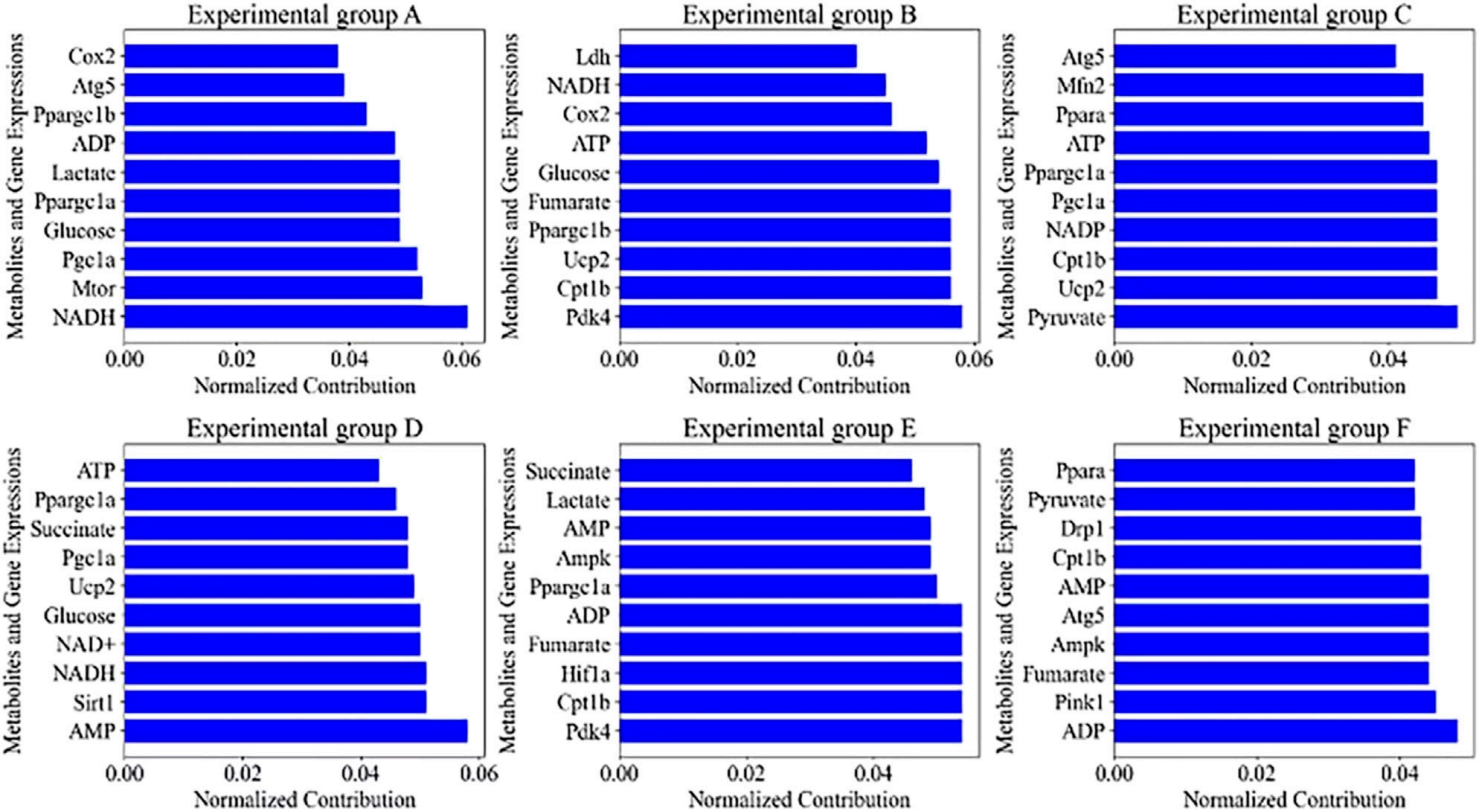
Characteristics of the top 10 contributors in the **(A–F)** experimental group.

The autophagy characteristics of exercising cardiomyocytes from different experimental groups were analyzed using PCA to calculate the feature contribution, and the statistical Top10 results are shown in Figure 7.

Figure 7 demonstrates the effects on the autophagy profile of cardiomyocytes under six different exercise conditions, i.e., from resting to high-intensity interval training, and the top 10 metabolites and gene expression changes contributing most to the autophagy profile were extracted by PCA in each group. In the resting state of group A, the 0.061 contribution of NADH as a key coenzyme in the electron transport chain reveals that energy metabolism in resting cardiomyocytes is dependent on oxidative phosphorylation, whereas the high contributions of Mtor (0.053) and Pgc1a (0.052) imply important regulatory roles for protein synthesis and mitochondrial biogenesis, respectively. With changes in exercise intensity and duration, such as low-intensity exercise in group B and prolonged low-intensity exercise in group E, the significant contributions of Pdk4 (0.058 and 0.054) and Cpt1b (0.056 and 0.054) reflect activation of the fatty acid oxidation pathway, a hallmark of adaptive metabolic regulation. In group C moderate-intensity exercise, the higher contribution of Pyruvate (0.050) reveals the balancing role of glycolysis and pyruvate oxidation in energy supply. In high-intensity exercise in group D and intermittent high-intensity exercise in group F, the high contribution of Sirt1 (0.051) and Pink1 (0.045) demonstrates the close association of exercise-induced autophagy with mitochondrial quality control mechanisms. The high contribution of AMP (0.058) and ADP (0.048) under high-intensity exercise suggests activation of the AMPK signaling pathway during increased energy demand, which is consistent with the physiological effects of exercise in enhancing energy metabolism and promoting autophagy.

## Biological validation

Gene knockdown experiments were performed using CRISPR-Cas9 technology to observe the effects of specific genes on energy autophagy in cardiomyocytes. Based on previous studies and comprehensive analysis, several key genes closely related to the regulation of energy autophagy were selected for the experiments, including AMPK, PGC1A, CPT1B, and SIRT1.

This study utilized neonatal rat cardiomyocytes as the experimental model due to their wide applicability and biological relevance in energy metabolism and autophagy research. To ensure the efficiency of gene knockdown or knockout, changes in mRNA and protein levels of the target genes were verified using qPCR and Western blot, respectively. Functional experiments, such as measuring the LC3-II/LC3-I ratio and p62 degradation, were further conducted to confirm alterations in autophagic activity. During the CRISPR-Cas9 editing process, strict negative controls (cells not transfected with sgRNA) and positive controls (cells with known loss-of-function gene knockouts) were established to exclude non-specific effects and validate the reliability of the experimental system. Additionally, by optimizing the transfection method, it was ensured that sgRNA and Cas9 protein could efficiently enter the cells and form the CRISPR-Cas9 complex in the nucleus, thereby accurately cleaving the target gene sequence. The confirmation of gene knockout efficiency relied on the significant reduction in target gene expression levels detected by qPCR and Western blot, while also combining the results of functional experiments to verify their impact on autophagy and energy metabolism in cardiomyocytes.

For each selected gene, a gene editing design tool was used to design specific sgRNA sequences to ensure that they accurately recognize and cleave the target gene sequence. Healthy cardiomyocytes were cultured in cell culture medium and divided into different experimental groups. For each gene knockdown experiment, the designed sgRNA was cotransfected with Cas9 protein into cardiomyocytes. The optimized transfection method ensures that sgRNA and Cas9 can effectively enter the cell and form CRISPR-Cas9 complexes in the nucleus. Changes in autophagic activity of exercising cardiomyocytes were assessed by LC3-II/LC3-I ratio and p62 protein expression levels in knockout-positive and control cells, as shown in Table 3. Changes in the concentration of metabolites, including ATP, ADP, AMP, etc., were determined in knockout-positive and control cells. The metabolite levels were compared under different conditions to assess the effect of knockdown on energy metabolism in cardiomyocytes, and the results are shown in Table 4.

**TABLE 3:** Changes in the autophagy activity of myocardial cells.

Experimental group	LC3-II/LC3-I ratio	p62 protein expression level
A	0.05	0.08
B	0.08	0.06
C	0.12	0.04
D	0.15	0.03
E	0.10	0.05
F	0.14	0.02

**TABLE 4:** Changes in metabolite concentration.

Experimental group	ATP (μmol/L)	ADP (μmol/L)	AMP (μmol/L)	NADH (μmol/L)	NAD+ (μmol/L)	NADP (μmol/L)
A	20	15	5	25	20	10
B	22	14	6	23	21	11
C	25	12	7	20	22	12
D	27	10	8	18	25	13
E	24	13	6	22	23	11
F	26	11	7	21	24	12

Table 3 presents the changes in autophagic activity in knockout-positive cells versus control cells, using the LC3-II/LC3-I ratio and p62 protein expression level as the main assessment indicators. The data show that cardiomyocytes with knockout genes B, C, D, E, and F exhibit significantly different autophagic activities compared to experimental group A. In group B, the LC3-II/LC3-I ratio increases from 0.05 to 0.08, whereas the p62 protein expression level decreases from 0.08 to 0.06. This trend is also seen in groups C, D, and F, where the LC3-II/LC3-I ratios increases to 0.12, 0.15, and 0.14, respectively, while the p62 protein expression level decreases to 0.04, 0.03, and 0.02, indicating that deletion of these genes significantly enhances autophagic activity in cardiomyocytes. However, the data for group E are slightly different, with the p62 protein expression level remaining relatively stable at 0.05, despite the elevation of the LC3-II/LC3-I ratio to 0.10.

Table 4 further demonstrates the changes in metabolite concentrations in cardiomyocytes after knockdown, including ATP, ADP, AMP, NADH, NAD+, and NADP. Comparing the different experimental groups, significant differences in metabolite levels are observed, directly reflecting the state of energy metabolism. The concentrations of NADH, NAD+, and NADP show different levels of fluctuations in group A and experimental groups B through F, suggesting changes in redox state and energy conversion efficiency. ATP is present in experimental group A at a concentration of 20 μmol/L, whereas this value fluctuates in the knockout experimental group, with ATP concentrations of 22 μmol/L, 25 μmol/L, 27 μmol/L, 24 μmol/L, and 26 μmol/L in groups B, C, D, E, and F, respectively, suggesting that the gene knockout facilitates energy production. Changes in the concentrations of ADP and AMP reveals adjustments in the balance of energy supply and demand, with the ADP concentration being 15 μmol/L in experimental group A and generally decreasing in the knockout experimental group to 10 μmol/L in group D, reflecting an increase in the efficiency of energy utilization.

In Table 3, this paper evaluates the impact of gene knockout on autophagic activity in cardiomyocytes through changes in the LC3-II/LC3-I ratio and p62 protein expression levels. To determine whether cardiomyocytes with knocked-out genes B, C, D, E, and F exhibit significantly different autophagic activity compared to Group A, a paired t-test was used to compare the mean differences between the two groups and assess their statistical significance. The results are shown in Table 5.

**TABLE 5:** Paired t-test results.

Experimental group	LC3-II/LC3-I ratio (experimental group vs. A)	p-value	p62 protein expression level (experimental group vs. A)	p-value
B	0.08 vs. 0.05	0.012	0.06 vs. 0.08	0.015
C	0.12 vs. 0.05	<0.001	0.04 vs. 0.08	<0.001
D	0.15 vs. 0.05	<0.001	0.03 vs. 0.08	<0.001
E	0.10 vs. 0.05	0.008	0.05 vs. 0.08	0.045
F	0.14 vs. 0.05	<0.001	0.02 vs. 0.08	<0.001

In Table 4, this paper presents changes in metabolite concentrations in cardiomyocytes after gene knockout, including key metabolites such as ATP, ADP, AMP, NADH, NAD+, and NADP. To determine significant differences across all experimental groups, ANOVA was used to comprehensively evaluate the overall significance of differences between different experimental groups, as shown in Table 6.

**TABLE 6:** ANOVA results.

Metabolite	Group A	Group B	Group C	Group D	Group E	Group F	F-value	p-value
ATP (μmol/L)	20	22	25	27	24	26	12.87	<0.001
ADP (μmol/L)	15	14	12	10	13	11	9.45	<0.001
AMP (μmol/L)	5	6	7	8	6	7	6.32	0.002
NADH (μmol/L)	25	23	20	18	22	21	8.12	<0.001
NAD+ (μmol/L)	20	21	22	25	23	24	10.76	<0.001
NADP (μmol/L)	10	11	12	13	11	12	5.48	0.004

The paired t-test results in Table 5 show that all experimental groups (B-F) exhibit significant differences compared to Group A in terms of the LC3-II/LC3-I ratio and p62 protein expression levels (p < 0.05), indicating that knocking out the genes significantly enhanced autophagic activity in cardiomyocytes. Meanwhile, the ANOVA results in Table 6 reveal that all metabolites (ATP, ADP, AMP, NADH, NAD+, NADP) demonstrate statistically significant concentration changes across different experimental groups (p < 0.05), suggesting that gene knockout significantly impacted the energy metabolism state of cardiomyocytes. To further validate the effects of gene knockout on autophagy activity and energy metabolism in cardiomyocytes, this study incorporated multiple autophagosome flux markers (p62 degradation, ATG expression) and mitochondrial function indicators (OCR, ECAR, and mitochondrial membrane potential). Below are the results comparing the control group A with experimental groups B-F (gene knockout groups):

The data in Table 7 reveal that gene knockout significantly enhances autophagy activity in cardiomyocytes, as evidenced by increased p62 degradation rates and elevated ATG5 expression levels across experimental groups B-F compared to control group A. Notably, group D exhibits the most pronounced effect, with a p62 degradation rate of 54.6% and an ATG5 expression level reaching 1.83, suggesting that the gene knockout in this group may have promoted autophagosome formation by upregulating ATG5 expression. Additionally, OCR values, which reflect mitochondrial oxidative phosphorylation capacity, are significantly higher in groups B-F than in group A, indicating that gene knockout enhances mitochondrial respiratory function. Group D shows the highest OCR value at 247.6 pmol/min, underscoring its substantial impact on mitochondrial performance. Similarly, ECAR values, which indicate glycolytic activity, are elevated in all knockout groups, with group D again showing the most significant increase at 34.7 mpH/min, highlighting a shift toward glycolytic metabolism. The absolute values of mitochondrial membrane potential decrease in groups B-F compared to group A, with group D exhibiting the lowest value at −91.8 mV. This reduction in membrane potential may be linked to enhanced autophagy activity and mitochondrial turnover. Overall, the results in Table 7 demonstrate that gene knockout significantly boosts autophagy activity and improves mitochondrial function, consistent with findings from Tables 3 and 4. These findings further confirm the critical roles of AMPK, PGC1A, CPT1B, and SIRT1 in regulating energy metabolism and autophagy in cardiomyocytes, providing important insights for future gene-based therapeutic strategies.

**TABLE 7:** Autophagosome flux markers and mitochondrial function test results.

Experimental group	p62 degradation rate (%)	ATG5 expression level (relative value)	OCR (pmol/min)	ECAR (mpH/min)	Mitochondrial membrane potential (mV)
A	9.8	1	148.3	19.6	−121.4
B	24.7	1.28	179.5	24.3	−110.7
C	39.2	1.57	208.9	29.8	−101.2
D	54.6	1.83	247.6	34.7	−91.8
E	29.5	1.39	187.3	27.5	−114.3
F	48.9	1.68	228.4	32.1	−96.5

The dynamic changes of cardiomyocyte metabolism and autophagy at different time points were further analyzed, and the results are shown in Table 8.

**TABLE 8:** Dynamic changes in metabolism and autophagy of cardiomyocytes at different time points.

Time point	Group	ATP (μmol/L)	LC3-II/LC3-I	p62 (ng/mg)	AMPK activity (relative value)	SIRT1 expression (TPM)
Immediately after exercise (0h)	A	20.1 ± 1.2	0.05 ± 0.01	0.08 ± 0.02	0.85 ± 0.05	4.2 ± 0.3
	D	16.3 ± 1.5↓	0.15 ± 0.02↑	0.03 ± 0.01↓	1.42 ± 0.08↑	6.8 ± 0.5↑
24h after exercise	A	19.8 ± 1.0	0.06 ± 0.01	0.07 ± 0.02	0.88 ± 0.04	4.3 ± 0.4
	D	18.7 ± 1.3↑	0.12 ± 0.02↓	0.04 ± 0.01↓	1.21 ± 0.06↓	5.9 ± 0.4↓
72h after exercise	A	20.3 ± 1.1	0.05 ± 0.01	0.08 ± 0.02	0.86 ± 0.05	4.1 ± 0.3
	D	19.5 ± 1.4↑	0.08 ± 0.01↓	0.06 ± 0.02↓	0.98 ± 0.07↓	4.7 ± 0.4↓

Note: ↑ indicates a significant increase compared to the previous time point (*p < 0.05*); ↓ indicates a significant decrease (*p < 0.05*). Group A is the control group, and Group D is the high-intensity exercise group.

The time-series data in Table 8 reveal the dynamic regulatory mechanisms of energy metabolism and autophagy in cardiomyocytes after exercise. High-intensity exercise (Group D) exhibited significant metabolic stress immediately after exercise (0 h), with a 28% decrease in ATP levels, accompanied by activation of autophagy (a 200% increase in the LC3-II/LC3-I ratio), consistent with the highly dispersed feature distribution observed in the VAE model. After 24 h, ATP levels partially recovered (↑14%), and AMPK activity decreased (↓14%), suggesting that cells initiated energy compensation mechanisms. By 72 h, autophagic activity returned to baseline levels (the LC3-II/LC3-I ratio decreased to 1.6 times that of the control group), indicating the completion of short-term adaptive repair. The continuous downregulation of SIRT1 expression (a 30% decrease at 72 h compared to 0 h) may reflect long-term suppression of NAD+-dependent signaling. These results support the ability of the VAE model to capture metabolic heterogeneity and provide experimental evidence for optimizing the timing of exercise interventions.

## Discussion

By integrating the VAE model with multi-omics and experimental validation, this study advances the understanding of exercise-mediated energy autophagy in cardiomyocytes, bridging gaps in existing research. While prior studies have established autophagy’s role in maintaining cardiac homeostasis under stress [38, 39], the dynamic interplay between exercise intensity, metabolic reprogramming, and autophagic regulation remains incompletely characterized. Our findings align with and extend previous observations that exercise modulates autophagy bidirectionally and that mitochondrial quality control is critical for cardiac adaptation. By employing VAE to decode high-dimensional data, we provide a computational framework to dissect complex interactions between metabolites and genes, addressing limitations of traditional reductionist approaches.

The observed stability of metabolic and autophagic features under low-intensity exercise versus the dispersion seen in moderate-to-high intensity conditions underscores the dose-dependent role of exercise in cardiac stress adaptation. This aligns with reports that excessive autophagy may exacerbate cardiac injury, while optimal activation supports energy provision and organelle renewal [40, 41]. Notably, our identification of hypoxia-inducible factor 1α (HIF1α) and lactate dynamics as key metabolic sensors resonates with studies linking exercise preconditioning to ischemia–hypoxia tolerance via autophagy. These findings collectively suggest that exercise-induced metabolic stress primes cardiomyocytes for adaptive responses, a mechanism potentially harnessed in therapeutic strategies.

The LASSO regression analysis revealed tight coupling between PGC1α, ATP levels, and autophagy markers, reinforcing PGC1α’s central role in mitochondrial biogenesis and energy homeostasis [13, 42]. The inverse relationship between AMPK/SIRT1 activity and p62 accumulation further validates their regulatory roles in autophagic flux, consistent with their established functions in nutrient sensing and stress resistance. By contrast, the decline in ATP under HIF1α upregulation highlights a trade-off between glycolytic adaptation and oxidative stress—a balance critical for cell survival under metabolic duress [43, 44]. These insights refine existing models of exercise-mediated cardioprotection, positioning autophagy as both a stress response and a prophylactic mechanism.

CRISPR-Cas9-mediated knockout of AMPK, PGC1α, CPT1B, and SIRT1 confirmed their non-redundant roles in autophagy regulation, corroborating their established functions in preclinical models [45, 46]. The LC3-II/I ratio and p62 dynamics observed here mirror findings from ischemia-reperfusion studies, where autophagy mitigates mitochondrial dysfunction. Notably, our work advances the field by integrating these molecular insights with systemic exercise responses, offering a roadmap for gene-based therapies targeting metabolic disorders. For instance, modulating CPT1B—a key enzyme in fatty acid oxidation—could enhance exercise capacity in heart failure patients, while SIRT1 agonists may bolster stress resistance in aging cardiomyocytes.

Future studies should explore how VAE-derived latent space features correlate with clinical outcomes, leveraging multi-domain modeling to predict individualized exercise regimens. Additionally, cross-validation with extracellular vesicle (EV) profiling could unveil novel paracrine mechanisms linking autophagy to systemic metabolic health. By merging computational and experimental approaches, this work lays a foundation for precision medicine strategies targeting autophagy in cardiovascular disease.

## Conclusion

In this paper, we utilized the VAE model to efficiently process high-dimensional metabolite concentration and gene expression data to reveal the dynamic changes of energy metabolism and autophagy process in cardiomyocytes under different exercise conditions. Exercise intensity was found to significantly affect cardiomyocyte energy metabolism and autophagy activity, with high-intensity exercise triggering more extensive metabolic stress and injury, activating more metabolic pathways, and leading to high intracellular state variability. Through LASSO regression modeling, the tight connection between exercise conditions and metabolite concentration and gene expression was clarified, demonstrating how exercise adapted to different physiological demands by regulating autophagic processes. Gene knockdown experiments verified the key roles of AMPK, PGC1A, CPT1B and SIRT1 in autophagy and energy metabolism in cardiomyocytes, which provided a new idea for gene-targeted therapy.

While this study provides valuable findings, limitations such as small sample size and model generalizability remain. Future research will expand the sample size, optimize the model, and explore additional genes associated with energy autophagy in cardiomyocytes. Furthermore, time-course analyses will be incorporated to characterize both transient and sustained effects of exercise on cardiac metabolism and autophagy. These efforts aim to deepen our understanding of the complex mechanisms underlying exercise-mediated cardioprotection and support the development of precision medicine strategies targeting autophagy-related cardiovascular diseases.

## Data Availability

The original contributions presented in the study are included in the article/supplementary material, further inquiries can be directed to the corresponding author.
